# Life cycle evolution: was the eumetazoan ancestor a holopelagic, planktotrophic gastraea?

**DOI:** 10.1186/1471-2148-13-171

**Published:** 2013-08-16

**Authors:** Claus Nielsen

**Affiliations:** 1 Zoological Museum, The Natural History Museum of Denmark, University of Copenhagen, Universitetsparken 15, DK-2100, Copenhagen, Denmark

**Keywords:** Larvae, Evolution, Adaptation, Planktotrophy, Gastraea, Trochaea, Dipleurula

## Abstract

**Background:**

Two theories for the origin of animal life cycles with planktotrophic larvae are now discussed seriously: The terminal addition theory proposes a holopelagic, planktotrophic gastraea as the ancestor of the eumetazoans with addition of benthic adult stages and retention of the planktotrophic stages as larvae, i.e. the ancestral life cycles were indirect. The intercalation theory now proposes a benthic, deposit-feeding gastraea as the bilaterian ancestor with a direct development, and with planktotrophic larvae evolving independently in numerous lineages through specializations of juveniles.

**Results:**

Information from the fossil record, from mapping of developmental types onto known phylogenies, from occurrence of apical organs, and from genetics gives no direct information about the ancestral eumetazoan life cycle; however, there are plenty of examples of evolution from an indirect development to direct development, and no unequivocal example of evolution in the opposite direction. Analyses of scenarios for the two types of evolution are highly informative. The evolution of the indirect spiralian life cycle with a trochophora larva from a planktotrophic gastraea is explained by the trochophora theory as a continuous series of ancestors, where each evolutionary step had an adaptational advantage. The loss of ciliated larvae in the ecdysozoans is associated with the loss of outer ciliated epithelia. A scenario for the intercalation theory shows the origin of the planktotrophic larvae of the spiralians through a series of specializations of the general ciliation of the juvenile. The early steps associated with the enhancement of swimming seem probable, but the following steps which should lead to the complicated downstream-collecting ciliary system are without any advantage, or even seem disadvantageous, until the whole structure is functional. None of the theories account for the origin of the ancestral deuterostome (ambulacrarian) life cycle.

**Conclusions:**

All the available information is strongly in favor of multiple evolution of non-planktotrophic development, and only the terminal addition theory is in accordance with the Darwinian theory by explaining the evolution through continuous series of adaptational changes. This implies that the ancestor of the eumetazoans was a holopelagic, planktotrophic gastraea, and that the adult stages of cnidarians (sessile) and bilaterians (creeping) were later additions to the life cycle. It further implies that the various larval types are of considerable phylogenetic value.

## Review

### Background

**Figure 1:**
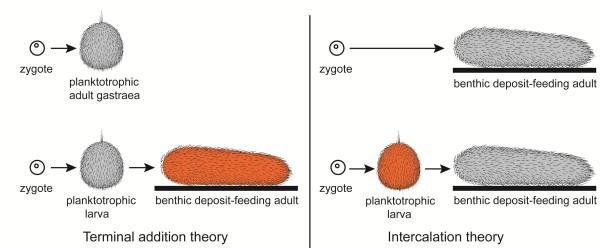
**Theories about the origin of the indirect life cycles.** The upper row shows the ontogenies of the ancestral organisms, and the lower row shows the ontogenies of the indirect, pelago-benthic organisms with the added life cycle stages indicated in red. The cnidarians have sessile adults instead of creeping adults.

**Figure 2:**
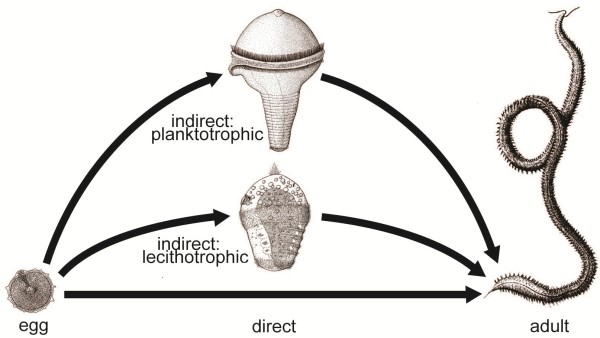
**Types of invertebrate life cycles.** The definition of direct versus indirect development is not precise (see the text), and there are a few “facultative feeding” larvae, which feed in the plankton if food is available, but which are capable to go through metamorphosis without feeding.

The origin of the indirect (pelago-benthic, biphasic) life cycle with a ciliated, planktotrophic larva and a benthic adult has been discussed for more than a century. Many authors have favored the idea that the planktotrophic larvae are ancestral in the (eu)metazoans, for example [1-4], whereas most of the recent authors believe that the feeding larvae are specializations of the ontogeny of an ancestral, direct development, for example [5-7] (see Figure 1). There are many cases of intermediates between indirect development with lecithotrophic larvae and direct development, so in the following I will discuss development with planktotrophic larvae versus development with lecithotrophic larvae or direct development (Figure 2 and Table 1).

**Table 1:** Glossary of technical terminology

**Phylogenetic terms**	**Developmental types**	**Larval types**
	(see also Figure 2)	
clade (monophyletic group) - an ancestor and all its living and extinct descendents	direct development - development without a larval stage	gastrula - hypothetic ancestor (gastraea) and early developmental stage of many neuralians, consisting of ecto- and endoderm
apomorphy (advanced character) - a new character (state) at a node on the phylogenetic tree	indirect development (also called biphasic life cycle, including pelago-benthic life cycle) - development with a larval stage	ephyra - newly strobilated scyphozoan medusa
		trochaea - hypothetic protostomian ancestor, a gastrula with an archaeotroch, i.e., a peri-blastoporal ring of compound cilia (see Figure 11)
		trochophora - typical larva of spiralians with downstream-collecting ciliary bands (see Figures 5 and 6)
plesiomorphy (primitive character) - a character (state) inherited from an earlier node on the tree	lecithotrophic - larva nourished exclusively by yolk	
		cyphonautes - planktotrophic larva of bryozoans with ciliary sieving
	planktotrophic - larva feeding in the plankton	actinotrocha - larva of phoronids with tentacles with ciliary sieving
	facultative feeding – larva which may feed in the plankton, but which can develop normally without feeding	dipleurula - typical larva of ambulacrarians with perioral band of single cilia with upstream-collecting by ciliary reversal; the larvae of the echinoderm classes have special names (see Figure 9)
homoplasy (convergence) - apparently similar structures evolved independently in different clades		
	poecilogony - various developmental types in the same species	tornaria - dipleurula larva of enteropneusts with a perianal band of compound cilia used in swimming (see Figure 9)

**Figure 3:**
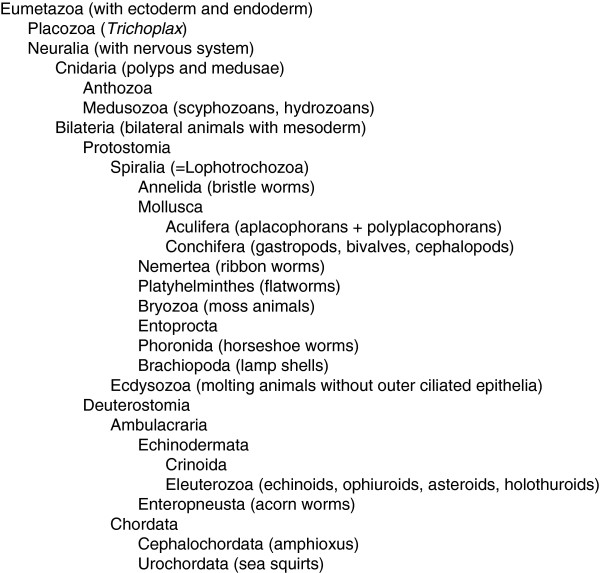
Taxonomic overview of higher animal groups mentioned in the text.

Haeckel’s famous gastraea theory [8] proposed that all metazoans have evolved from a pelagic, planktotrophic ancestor called gastraea, and this implies that the benthic adult stages have been added to the life cycle in one or more lineages. This has been called the terminal addition theory. A planktotrophic gastrula stage is found in a number of anthozoans, and non-feeding gastrula stages are found in representatives of almost all phyla. In lineages with indirect development, the feeding larvae have become modified to the many different larval forms we see today. The terminal addition theory has sometimes, quite self-contradictory, been called the larva-first theory. More precisely, Hatschek [9] pointed to the similarities between some rotifers and the trochophora larvae of annelids and molluscs and proposed that the ancestor of the Protostomia (which he called Zygoneura) (Figure 3) was a holopelagic, planktotrophic gastrula-like organism called protrochula; the indirect development evolved when a benthic stage was added and the planktonic stage was modified into a trochophora larva. This seems to be the first well-founded scenario for the evolution of an indirect life cycle.

An alternative theory interprets the various planktotrophic larval types as numerous independent deviations from an ancestral direct development of a planuloid ancestor; this has been called the intercalation or interpolation theory, for example [5,10]. A variation of the intercalation theory, viz. that the planktotrophic trochophores are evolved from non-feeding larvae with a prototroch through addition of metatroch and adoral ciliary zone, is favored by a number of authors [11-13]. The homology of spiralian prototrochs seems unquestioned, and the origin of metatroch and adoral ciliary zone of the ciliary filter feeding trochophores is discussed below.

The morphology of the (eu) metazoan ancestor is of course of great importance for the discussion, and a number of more or less realistic ideas have been put forward over the last one and a half century. Some earlier theories, such as the cellularization or ciliate-acoel theory, envisaged the evolution of a planula-like metazoan ancestor from a ciliate-like ancestor which became cellularized [14,15]. However, the molecular phylogeny has clearly shown that the ciliates are far from the metazoans on the tree of life [16]. Parts of this theory survive with modifications imbedded in some variations of the planula theory (see below). Other theories, such as the “biphasic life cycle theory” [17], the “complex bilaterian ancestor theory” [18], and the “clonal asexual reproduction theory” [19] propose the origin of the bilaterian phyla from budding, colonial ancestors, but this finds support neither from morphology nor from molecular phylogeny. I will not pretend that I understand the early stages of the “synzoospore theory” [20], but the late evolutionary steps leading to the eumetazoan ancestor through specialization of a blastula to a gastrula and the loss of the sessile, filter-feeding adult through “neoteny” is almost identical to the process proposed by me [21], except that I now call the evolutionary process “truncation” instead of neoteny [22]. The “plakula theory” [23] proposes the specialization of the epithelium of a blastaea into a lower entoderm, used in feeding and locomotion, and an upper ectoderm; it can be seen as a variation of the gastraea theory. Here it should be emphasized that gastrulation is related to the separation of a digestive epithelium from a protective/locomotory epithelium, not just the creation of a multilayered organism as suggested by some authors [24]. The gastrula consists of the archenteron lined by the digestive endoderm, surrounded by the locomotory ectoderm. The placozoan *Trichoplax* creeps on the digestive epithelium [25], but this is probably a specialization. A further specialization is seen in the parasitic hydroid colonies of the hydrozoan *Polypodium*, which live turned inside-out in sturgeon’s eggs with the digestive endoderm on the outside and small polypides inside the ectodermal invagination [26].

Only the gastraea/terminal addition and planula/intercalation theories are now more generally accepted, and they will be discussed in the following.

Several types of information have been used to infer ancestral life cycles, and it is important to consider all information when making inferences. However, it appears practical first to discuss the types of information separately.

This review will deal with the life cycle evolution of marine invertebrates with focus on phyla with ciliated “primary” larvae, i.e. Cnidaria, Spiralia and Ambulacraria. Ecdysozoa and Olfactores (Urochordata + Vertebrata) lack ciliated larvae and their larval stages are “secondary”, as are the nauplius larvae and the caterpillars [2,3,27]. The early cephalochordate larva is a ciliated, non-feeding gastrula.

### Discussions

#### Evidence from the fossil record

The only unequivocal information about evolution comes from the fossil record, and Late Precambrian and Early Cambrian fossils have been studied for evidence about the ancestral developmental type of the eumetazoans. Direct evidence about evolution from indirect to direct development or vice versa can be gathered from information about evolution of developmental types in later, well-known fossil lineages.

Microscopic embryos and larvae are usually without fossil parts and are therefore not likely to fossilize, but a few Ediacaran (Latest Precambrian) fossils have been interpreted as metazoan embryos. The small, spherical acritarchs from the Earliest Ediacaran (Doushantuo Formation) could be metazoan eggs with an elaborate egg membrane, but they could just as well be cysts of various algae [28,29]. Globular fossils with a diameter of about 500 μm from the Doushantuo Formation have been interpreted as early cleavage stages of metazoan eggs [30,31], but they have also been interpreted as giant bacteria [32], and new studies have concluded that the internal structures are incompatible with those of metazoan eggs and embryos [33].

Out of many thousand small globular fossils with a diameter of up to 200 μm from the Doushantuo Formation, a few, called *Vernanimalcula*, show internal structures which have been interpreted as surprisingly well-preserved bilaterian embryos with gut, paired coelomic sacs and a number of sensory organs, which should have been preserved through mineralization deposited on decaying tissues [34-36]. However, the original interpretation has been questioned, and a recent comparison of the original description with sections of decaying cyst-forming protists and acritarchs of similar age concluded that the “anatomical reconstruction [of *Vernanimalcula* as a bilaterian] is without foundation” [37]. Putative cnidarian larvae from the same deposits [38] are of the same nature. So there is no direct information about the developmental type of the earliest eumetazoans.

*Olivooides* (first based on embryos, the adults were called *Punctatus*) from the slightly younger Dengying Formation through Lower Cambrian [39-41] have been interpreted as stem-group cnidarians. The adults resemble the fossil conulariids and the scyphistoma polyps of living coronate scyphozoans, but show a pentameric symmetry. However, the similar, co-occurring *Quadrapyrgites* is tetraradial. The development from the about 500 μm-diameter egg to juvenile is clearly direct, and the new observations indicate that a strobilation process was present, indicating scyphozoan affinities, and putative ephyrae were also observed.

A considerable number of Ediacaran (Vendian) fossils have been interpreted as bilaterians [42], but the metazoan nature of several forms has been questioned. *Dickinsonia* has been interpreted as a placozoan [25], and *Kimberella* as a mollusc [43]. None of these fossils give information about type of development.

Representatives of the majority of Living metazoan phyla appear in the fossil record in the Cambrian, in the Early Cambrian (Atabdanian) Sirius Passet and Chengjiang faunas [44] and in the Middle Cambrian Burgess Shale [45]. These old faunas comprise many soft bodied organisms, but only the fossils with hard skeletons have left information about developmental types. There is a well proven correlation between egg size and developmental type, with small eggs (usually about 100 μm or less in diameter) developing into planktotrophic larvae, whereas larger eggs usually develop into non-feeding stages [46], so the sizes of larval shells can give information about developmental types.

**Figure 4:**
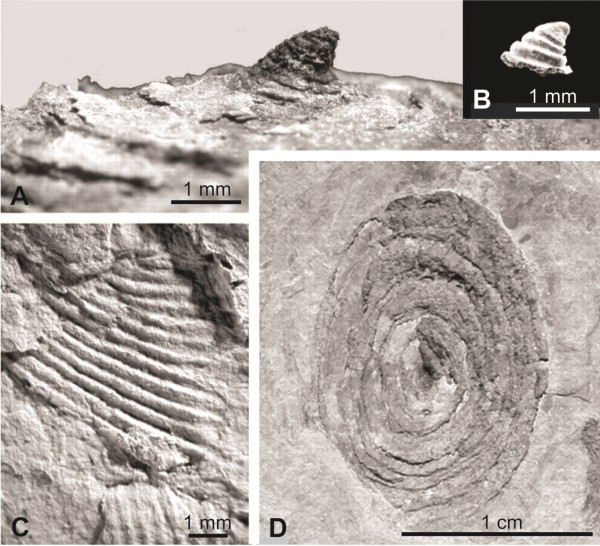
**Larval and adults shells of Lower Cambrian helcionellids. A**, Apex of a centimeter-large adult showing the larval shell. **B**, A “small shelly fossil” showing the exact same morphology as the apex of the adult helcionellid. **C**, Detail of the sculpture of the adult shell. **D**, A whole fossil helcionellid. Modified from [48].

“Small shelly fossils” appear with increasing diversity through the Earliest Cambrian (Nemakit-Daldynian and Tommotian) and decline in the Middle Cambrian. They are mostly interpreted as molluscs, but a few as brachiopods and a number as extinct groups of uncertain affinities [47]. Most of them are scales or spicules of larger animals and give no information about developmental types. However, a millimeter-sized, cap-shaped fossil, usually interpreted as an early helcionellid conchiferan helcionellid mollusc, has now been found forming the apex of a centimeter-sized limpet-like fossil, and this indicates that the organism was a stem-lineage gastropod with a planktotrophic larva [48] (Figure 4).

The Mollusca originated in the Late Precambrian [49], and there is no indication of an ancestral form with a shelled larva. Fossils of gastropods and bivalves are known from the Early Cambrian [50] (and see above). Gastropods, bivalves and some extinct forms retain larval shell(s) at the apex/umbo, which give indications about the type of larva. In Living species shells with a small embryonic shell (protoconch 1/prodissoconch I; the shell formed before the embryo hatches from the egg membrane) and a large larval shell (protoconch 2/prodissoconch II; formed during the planktotrophic phase), is characteristic of species with planktotrophic development. Species with direct development have a large embryonic shell and lack a larval shell [51]. There is much discussion about the developmental type of the Cambrian molluscs, see for example [52-54]. Gastropoda probably originated in the Late Cambrian, and it appears that many of the early forms, including the helcionellid mentioned above, had larval shells and therefore planktotrophic development [55]. The Early Cambrian bivalve *Pojetaia* had an embryonic shell which is large for a planktotrophic species (about 100 × 150 μm), but the size of the putative larval shell (about 300 μm) indicates the presence of a planktotrophic larva [56].

Studies of evolution of later gastropod groups with analyses of larval types are scarce, but an analysis of protoconchs of six families of fossil “neogastropods” from Early Tertiary of the Gulf of Mexico area revealed a low proportion of species with large protoconchs in the Early Palaeocene with increasing numbers into the Eocene, indicating evolution of direct development [57]. Evolution of non-planktotrophic development from planktotrophic development was observed in a number of genera, such as *Athelata, Agaronia*, and *Latirus*, whereas evolution in the opposite direction was not observed.

Brachiopods can be traced back to the Early Cambrian, and the size of the larval shells of some of the earliest fossils indicate that the stem lineages of both Linguliformia and Rhynchonelliformia had planktotrophic larvae [58].

Echinoderms have a very extensive fossil record, with non-pentameric stem groups in the Cambrian [59], but none of these old fossils show any indication of their developmental type.

**Figure 5:**
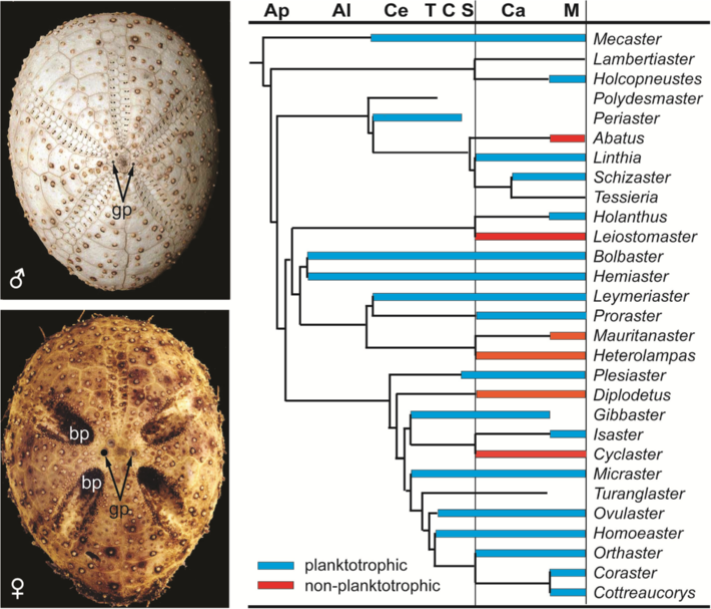
**Developmental types of spatangoid echinoids.** Left photos: Tests of a male and a female of the echinoid *Brachysternaster chesheri*. The brood pouches (bp) in the female and the difference in gonopore size in the two sexes are seen (gp; the male test is cleaned, whereas the female still has some of the organic material partially covering the gonopores; the black dot at the left side is inserted to indicate the size of the clean gonopore). Photos from http://www.nhm.ac.uk/research-curation/research/projects/echinoid-directory/taxa/taxon.jsp?id=429. Right diagram: Evolution of developmental types of Cretaceous spatangoid echinoids. Only the period from the Aptian to the Maastrictian is shown, but seven successive outgroups from the Valanginian to the Aptian all had pluteus larvae. Ap, Aptian; Al, Albian; Ce, Cenomanian; T, Turonian, C, Coniacian; S, Santonian; Ca, Campanian; M, Maastrichtian. Modified from [60].

The developmental type of fossil echinoids can in some cases be inferred from three types of structures [60] (Figure 5): 1) Presence of brood pouches indicate direct development, 2) Extreme dimorphism of gonopore size indicate the presence of large eggs and therefore non-planktotrophy, and 3) Crystallographic orientation of genital plates: At metamorphosis of the planktotrophic echinopluteus larvae, the basal part of the calcareous skeletal rod of four of the large arms become retained in four of the genital plates, and they show a crystallographic orientation different from that of the remaining genital plate and of the other test plates. Species with direct development show identical crystallographic orientation in all plates [61]. These three methods were applied to a large material of Upper Cretaceous spatangoids, and was clearly shown that the planktotrophic type of development was ancestral in the family and that non-planktotrophy evolved independently five times during the Campanian-Maastrichtian period (Figure 5), possibly related to environmental changes [60]. No brooding or lecithotrophic echinoids have been found in older deposits [62].

The Precambrian/Early Cambrian fossils contain no unambiguous information about the ancestral developmental type of the eumetazoans. The earliest gastropods and bivalves probably had planktotrophic larvae. Observations on fossils from later periods contain well-documented examples of evolution of groups with non-planktonic life cycles in lineages with planktotrophic development, but apparently no examples of evolution in the opposite direction.

So, the fossil record indicates that the indirect development with planktotrophic larvae was ancestral.

#### Mapping of developmental types onto known phylogenies

**Figure 6:**
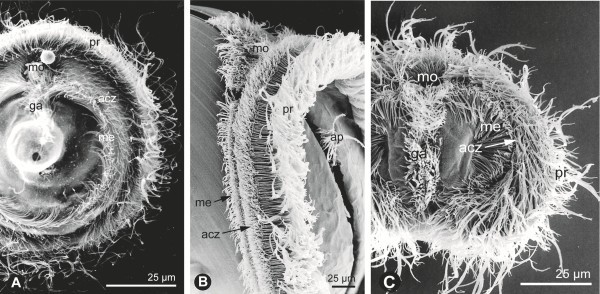
**Downstream-collecting ciliary complexes of trochophora larvae, SEM. A**, The annelid *Serpula oregonensis.***B**, The bivalve *Barnea candida*. **C**, The entoproct *Loxosomella elegans*. From [22].

**Figure 7:**
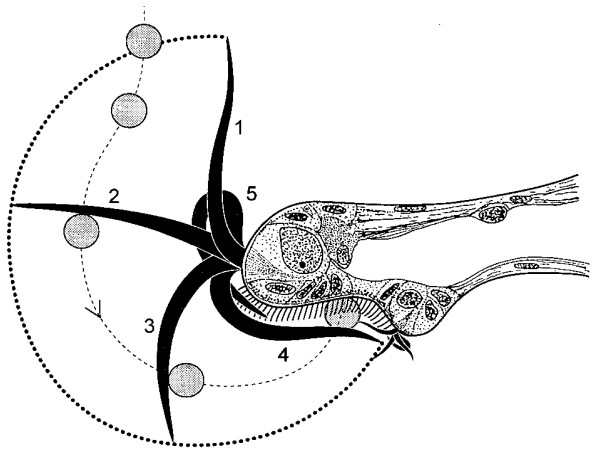
**The catch-up principle.** Diagram of a cross-section of the velar edge of the gastropod *Crepidula fornicata.* The thick, compound cilia of prototroch and metatroch beat towards the band of single cilia of the adoral ciliary zone (food groove). The sequence of stages of the prototroch cilia are indicated by the small numbers. The prototroch cilia cut through the water, catch up with a food particle which is then pushed on to the adoral ciliary zone. This is apparently aided by the beat of the metatroch cilia. These cilia of the adoral zone carry the particles towards the mouth. From [66].

The idea behind this method is that the distribution of different developmental types should make it possible to deduce the developmental types of ancestors of increasing age, and it has been used in cladistic analyses for example of polychaetes [13,63-65]. However, the method builds on the assumption that gains and losses have the same weight, and this is obviously not the case for complicated structures, such as the highly specialized ciliary bands of trochophora larvae (Figure 6) which function as downstream-collecting structures based on the “catch-up principle” [66] (Figure 7). As shown below, larval structures can be lost through the silencing of just one gene (see the section “Genetics-related information”), and the evolution of the complicated ciliary bands of a trochophore from a uniformly ciliated larva has no adaptations-based explanation (see the section Scenarios below). So the method has apparently only little credibility when applied to larger groups, but it has been used on a number of smaller clades, and a few examples will be discussed below to demonstrate some of the pitfalls of the method.

Ecdysozoans lack ciliated outer epithelia completely (and therefore also primary larvae), and this is unquestionably an apomorphy [6]. Cephalochordates have ciliated non-feeding larvae, but Urochordata + Vertebrata (=Olfactores) lack ciliated primary larvae. The remaining major eumetazoan (neuralian) clades have characteristic types of ciliated, feeding larvae: cnidarians have planula larvae, spiralians trochophora larvae, and ambulacrarians dipleurula larvae.

The cnidarian larva is usually called planula, a name originally given to the compact larva of the hydroid *Eudendrium rameum*[67]. The term is now used for all types of cnidarian larvae, but also more widely for small compact lecithotrophic larvae, which is unfortunate because several anthozoans, such as the actinian *Anthopleura*[68] and the solitary corals *Caryophyllia* and *Fungia*[69,70], have feeding gastrula larvae. The anthozoans are apparently the basal group since some recent studies indicate that Medusozoa is an ingroup of the Anthozoa [71], so the feeding larva could well be ancestral.

Several spiralian phyla have representatives with indirect development with planktotrophic larvae. Hatschek [72] proposed the name trochophora in a general discussion of annelid development. In his famous, unfortunately unfinished “Lehrbuch” [9] he gave a more thorough discussion of the concept with special emphasis on nervous system and ciliary bands. He proposed that the trochophore was the larva of the ancestor of the Protostomia (Zygoneura). His seminal idea has been elaborated and modified by many subsequent authors, for example [73] and modified into the trochaea theory [74,75] (see section “Scenarios” below). Trochophora larvae are characteristic of most spiralian phyla, but the larvae of bryozoans, phoronids and brachiopods are different and all the types will be discussed below.

Annelida, including Echiura, Sipuncula and Pogonophora, is now a well-documented clade [76], but unfortunately, its phylogeny is still not firmly resolved. Occurrence of planktotrophic trochophora larvae has been reported in a number of families: Oweniidae [77,78], Serpulidae [79], Polygordiidae [72], Sabellariidae [80], Opheliidae [81], Echiuridae [81], Amphinomidae [3,82], Spionidae [83], and Capitellidae [84]. These families do not group together in any of the phylogenetic studies. Only very few of the planktotrophic annelid larvae have the telotroch, but all the ciliary bands considered as ancestral in the spiralians are found for example in the larvae of *Polygordius* and *Echiurus*[72,85], and the telotroch is found in many of the lecithotrophic larvae [13]. The occurrence of planktotrophic trochophora larvae in so many, not closely related polychaete families makes independent evolution of this larval type from non-feeding ancestors highly improbable. The cladistic analyses of Rouse [13,63,65] came to the conclusion that multiple evolution of the feeding larvae was the more parsimonious explanation. However, not all the just-mentioned families were included, and as mentioned above and discussed in the section “Scenarios” below), I don’t think that cladistic analyses are useful for characters where loss and gain are obviously not equally probable. In addition, a number of species show a remarkable plasticity of developmental types (see also poecilogony in the section “Genetics-related information” below).

Many serpulids, such as *Spirobranchus, Serpula, Hydroides,* and *Pomatoceros*[79,86] have almost schematic, filter-feeding trochophora larvae, whereas members of the sister-family Sabellidae apparently all have lecithotrophic larvae. However, the larva of the sabellid *Schizobranchia insignis*[87] is non-feeding but nevertheless develops the characteristic trochophore ciliary system, which even captures particles and transports them towards the mouth where they are rejected as the gut is not yet fully formed. This must be interpreted as a loss of the feeding function in connection with an increased amount of yolk, because the evolution of a complex structure as the ciliary feeding system without function has no adaptational value until complete (see section “Scenarios” below).

Thus, there is a considerable variation in polychaete development, but no pattern can be recognized indicating a de novo evolution of feeding larvae from ancestors with non-feeding larvae. The early radiation of the molluscan classes is still under discussion. Most recent studies of both the rich fossil record and the sequence data favor the monophyly of Aculifera and Conchifera [88]. It seems probable that the ancestral mollusc had no larval shell and that the shelled larva is a conchiferan apomorphy. However, the indirect life cycle with a ciliated larva or embryos with a prototroch and in some cases a metatroch or a telotroch is predominant in all the classes except the Cephalopoda and must therefore be considered ancestral.

**Figure 8:**
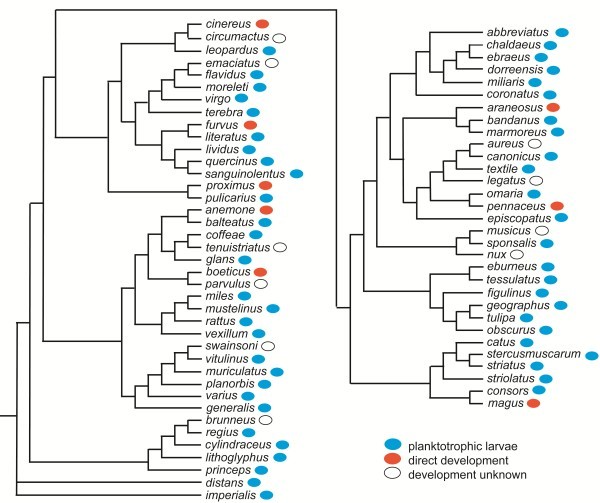
**The occurrence of “direct” development in the gastropod genus *Conus*.** Redrawn from [90]. The original paper distinguishes between species with planktonic larvae and non-planktonic development. Dr Alan Kohn (University of Washington) has informed me that all the planktonic larvae are planktotrophic.

**Figure 9:**
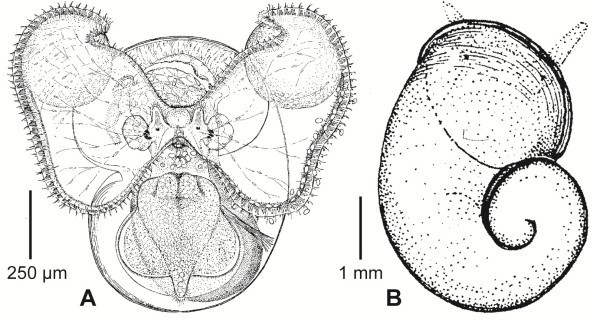
**“Direct” development in the gastropod *Cassidaria *sp. A**, The fully differentiated veliger larva inside the cocoon collects yolk particles with the ciliary bands of the large velum and transports them to the mouth. **B**, The newly hatched juvenile has lost all traces of the velum. Modified from [96].

Only gastropods and bivalves have representatives with planktotrophic larvae. These larvae are called veligers because the locomotory and particle-collecting ciliary system of the trochophore is situated on a smaller or larger expansion called the velum. Of the major gastropod lineages Neritimorpha, Caenogastropoda and Heterobranchia have representatives with feeding veliger larvae, whereas the remaining two groups, Patellogastropoda and Vetigastropoda, only comprise species with non-feeding larvae with a prototroch [89]. Analyses of smaller lineages, such as families or genera, for example the large genus *Conus*[90] (Figure 8), the family Littorinidae [91,92], the family Turritellidae [93] and the family Calyptraeidae [94,95] strongly indicate that direct development has evolved independently a number of times within these clades. Only the genus *Lacuna* within the Littorinidae show indication of evolution of planktotrophic species from direct developing ancestors. However, the term direct development is highly ambiguous when applied to gastropods. The development within the egg capsules may show embryos with fully differentiated veliger larvae, such as in *Cassidaria* (now *Galeodea*) sp. [96] where the ciliary apparatus at the edge of the velum transports yolk particles to the mouth; later stages in its development within the egg capsule show complete resorption of the velum, so that the hatching juvenile looks as a small adult (Figure 9). This is classified as direct development because the first free stage is a juvenile, but the change from planktotrophic development to “direct” development has not been a change in morphology, but only a change in the amount of yolk and a postponement of hatching from the egg capsule. A fossil of this type would undoubtedly be classified as an indirect developer. It seems to be a common feature of “direct” developing gastropods to have a more or less differentiated velum during the intracapsular development [96,97]. The term “larva” is usually defined as the stage from hatching to metamorphosis, but as mentioned for example by Hadfield (in [36]), this definition is more related to methods of dispersal than to changes in morphology; in most “direct-developing” gastropods, the morphological metamorphosis, with the loss of a velum or only of a prototroch, occurs before hatching from the egg capsule. If one would define the breaking of the fertilization membrane as the “hatching”, one could typify the above-mentioned gastropod veligers as intracapsular larvae (or “veliger-stage embryos” as suggested by Hadfield), but making definitions to cover all animals seems impossible [98].

The Bivalvia comprises the sister groups Protobranchia and Autobranchia [99], where the protobranchs have lecithotrophic pericalymma larvae and the autobranchs veliger larvae, which with few exceptions are planktotrophic [100]. The three bands of compound cilia on the early larva of the scaphopod *Dentalium (Antalis)* have the cell-lineage of a prototroch, so the very similar ciliary bands on the larval serosa of the protobranch *Acila* is very probably a prototroch too [101].

The prototrochs of the molluscs with ciliated larvae or embryos all have the same cell-lineage and structure as those of all the other spiralians, so the homology of these ciliary bands can hardly be questioned. Further, it seems most likely that feeding veliger larvae are ancestral in Gastropoda and Autobranchia, but the ancestral larval type of the molluscs cannot be deduced directly.

Entoprocts all have indirect development with more or less modified trochophora larva, which are feeding in most species [102,103].

Bryozoans all have indirect development. The planktotrophic cyphonautes larva is found in basal lineages of the Eurystomata [104,105], and the fossil record indicates that the Stenolaemata (with the Living Cyclostomata) is an ingroup of the Eurystomata [106,107]; so the cyphonautes may well have been the ancestral larval type. The corona of the lecithotrophic larvae could be a modified prototroch, but the bryozoans do not show spiral cleavage. The origin of the ciliated ridge, with a ciliary sieving system on multiciliate cells similar to that on the tentacles of the polypides [108], is enigmatic. The particle capture mechanism has earlier been thought to be similar to that of the echinoderm larvae, i.e. an upstream system with local ciliary reversal, but new observations have shown that the particles are captured by the laterofrontal cilia, which may function as a sieve and make flicking movements [109-111].

Almost all phoronids and the linguliform brachiopods have indirect development with feeding “larvae”. The planktonic linguliform larvae are swimming juveniles, essentially having the anatomy of the benthic adults with ciliated tentacles having a ciliary sieving system on monociliate cells. Particles become captured by laterofrontal cilia which either make a flicking movement or elicit a tentacle flick, in both cases moving the particle closer to the water current towards the mouth [112,113]. The actinotrocha larvae of the phoronids has tentacles of the same structure and function as those of the brachiopods, and the larval tentacles are retained as adult tentacles in some species [112,114]. The origin of this ciliary feeding mechanism has not been elucidated.

**Figure 10:**
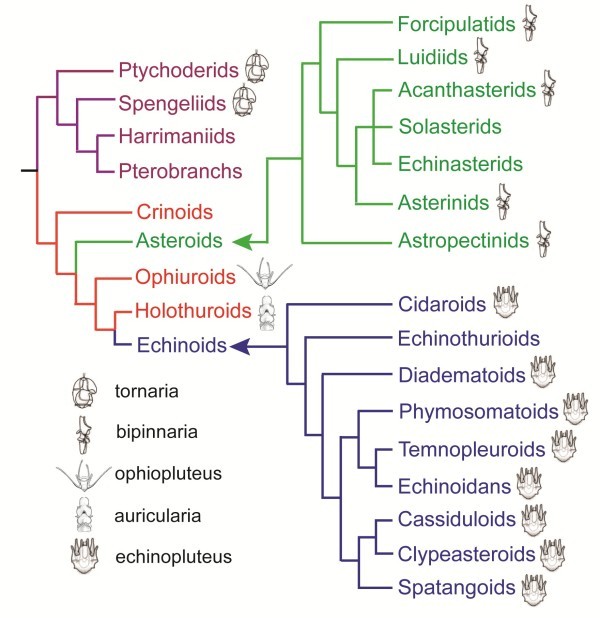
**Occurrence of planktotrophic larvae in ambulacrarian clades.** The small icons indicate the occurrence of the planktotrophic larval type in some species within the clade. Modified from [115].

Several studies have concluded that the feeding dipleurula is the ancestral larval type of echinoderms and enteropneusts (Ambulacraria) [115,116] (Figure 10). The planktotrophic larvae have a circumoral ciliary band (neotroch) of a unique structure and function: the band consists of monociliary cells performing upstream-collecting of particles based on ciliary reversal [117].

The radiation of the living echinoderm classes now seems well understood with Crinoidea as the sister group of the remaining classes (Eleutrerozoa) [118]. Feeding dipleurula larvae of various shapes are known from all the eleutherozoan classes (Figure 10). Feeding larvae have not been reported from any crinoid, but the sea lily *Metacrinus* has a lecithotrophic larva with a neotroch in the same shape as that of many of the early larval stages of the eleutherozoans; this ciliary band becomes reorganized into four circular bands through a process which is very similar to that observed in the initially planktotrophic larva of the holothurians *Synapta* and *Stichopus*[119,120]. Several studies of developmental types of smaller lineages demonstrate that lecithotrophic or direct development has evolved independently many times [121], for example in the echinoids [122] and in the family Asterinidae [123,124]. An evolution of a planktotrophic larval type from a non-feeding type has never been observed. This is in full agreement with the information from the fossil record mentioned above.

The neotroch of feeding enteropneust tornaria larvae is of the simple shape of a dipleurula in the early larvae, but becomes highly complicated during development in some species, especially in the Ptychoderidae. It is the first ciliary band to develop at the young larvae, and is initially used both in particle collection and swimming. The large perianal ring of compound cilia, which is the only ciliary band of the direct developing larvae, develops a little later [125].

Thus, there are many well-documented examples of evolution of non-planktotrophic development (lecithotrophic or direct) in all the phyla where ciliated planktotrophic larvae occur. The intercalation theory implies hundreds of examples of parallel evolution of planktotrophic trochophora larvae from non-feeding larvae, but no example of evolution from “direct development” to development with planktotrophic larvae. So, the tendencies are clearly in favor if the terminal addition theory.

#### The apical organ: The ancestral eumetazoan (neuralian) brain?

Almost all pelagic ciliated eumetazoan (neuralian) larvae have a group of nerve cells with long cilia at the apical pole. This organ has traditionally been called the apical organ, and this is unambiguous in cnidarian and ambulacrarian larvae. Unfortunately, the homologous organ in spiralian larvae is in many species intimately connected with a pair of lateral ganglia to form a compound organ which has also been called an apical organ. To avoid confusion, I have in previously tried to introduce the name “apical ganglion” for the organ [22,126,127], but it is not a real ganglion, and it appears that almost all recent papers use the term apical organ in the strict sense, so I have decided to revert to the old practice, following the recommendations of [128]. But it must be remembered that almost all older papers use the term for the compound organ.

The homology of cnidarian and bilaterian apical organs have been questioned, especially because the apical-blastoporal axis and the animal-vegetal axis have the same orientation in the bilaterians, but opposite orientations in the cnidarians, i.e., the polar bodies are situated at the apical pole in bilaterians but at the blastoporal pole in cnidarians. However, it now appears that gene expression supports the homology both of the apical organs and of the gastrulation areas and that the position of the polar bodies (and thus of the an-veg axis) may have changed in the bilaterians [129].

An apical organ is found in almost all cnidarian larvae. It comprises flask-shaped sensory cells and degenerates when the larva settles with the apical pole [130], in some species through apoptosis (personal communication from Dr Heather Marlow, EMBL Heidelberg).

Ciliated spiralian larvae almost all have an apical organ, with the cerebral ganglia developing almost simultaneously, often in close apposition to the apical organ. A close connection between their cerebral commissure and neurites from the basal parts of the apical cells are usually found. The apical organ degenerates before or at metamorphosis in all species [75,101,131], in some species through apoptosis [132].

Among the ambulacrarians, the enteropneust tornaria larvae have a conspicuous apical organ, which degenerates after metamorphosis [125]. Echinoderm larvae show considerable variation in the nervous organs at the apical pole. The early larvae of the eleutherozoan classes develop a small group of serotonergic sensory cells in more or less close contact with the apical loops of the ciliary band (neotroch); their homology with other apical organs appears uncertain [116]. The organ is apparently lost together with the ciliary bands at metamorphosis. The crinoid larvae develop a conspicuous ciliary tuft, but the organ resembles those of the eleutherozoan larvae, and the whole larval nervous system is lost at metamorphosis [133].

It appears that the apical organs are associated with the pelagic part of the life cycles and that they always disappear before or at metamorphosis. Thus, it fits well with the terminal addition theory, which proposes a planktonic gastraea with an apical organ as the ancestor of the eumetazoans (neuralians) [22]. There seems to be no indication of the existence of an apical organ in a direct developing ancestor, and a convergent evolution in many eumetazoan lineages appears highly unlikely.

#### Genetics-related information

Information about the genes involved in the organization of larval ciliary feeding bands would be of great importance for the understanding of their evolution. Are the same genes responsible for example for the development of the neotroch of all dipleurula larvae, and could a silencing of one or more of these genes lead to the uniformly ciliated, lecithotrophic larvae seen in several species? Unfortunately, there are only very few studies of this type. Comparisons of gene expressions in trochophora and tornaria larvae are of questionable value because of the possibility of homoplasy/homocracy [2,5,134].

The only comparative studies of ciliated larvae of closely related species with different life cycles appears to be observations on the larval stages of the indirectly developing echinoid *Heliocidaris tuberculata* with a normal echinopluteus larva and the direct developing *H. erythrogramma* and *Pseudoboleta maculata,* which have ovoid, uniformly ciliated larvae without a functioning gut. Fertilizing of eggs of *H. erythrogramma* with sperm from the about 4-million years distant *H. tuberculata* resulted in short-armed, pluteus-type planktotrophic larvae which went through a normal metamorphosis [135,136]. Experiments with fertilizing eggs of *H. erythrogramma* with sperm from the about 40-million years distant *P. maculata* resulted in very similar larvae, and it was concluded that their ancestral common species had indirect development with an echinopluteus larva [135]. This could indicate that the gene(s) governing the development of the pluteus arms and the larval gut are silenced in the direct developing species but can be partially activated by genes from the sperm of the indirect developing species (as in the ascidians, see below).

The later study of gene expression in gastrula and early pluteus stages of *H. tuberculata* and a stage of *H. erythrogramma* with developing primary tube feet [137] compares so disparate developmental stages that it must be considered as uninformative.

Observations on development of ascidians have shown that tailless (anuran) larvae have evolved several times within *Molgula* and *Pelonaia*[138] and that absence of *Manx* expression results in the anuran development [139]. This is clearly an example of a gene which regulates a switch between elongation and non-elongation of the cell groups which form the tail but which also later on develop into the characteristic chordate structures.

**Figure 11:**
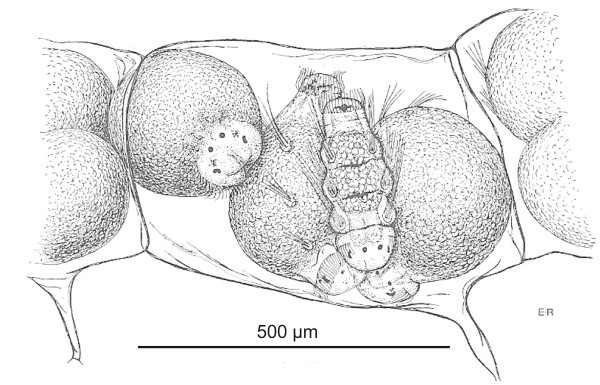
**Single egg capsule of the polychaete *Pygospio elegans.*** The capsule contains one “intracapsular trochophore” ready for hatching as a pelagic larva and three embryos full of yolk and almost ready for hatching as small juveniles. From [142] with permission from Taylor & Francis Ltd. http://www.tandfonline.com.

More circumstantial evidence comes from studies of poecilogony, i.e. the presence of different developmental types in the same species, for example in Annelida and Mollusca. In the annelid family Spionidae, *Streblospio benedicti*[140] two morphs are found one of which deposits small eggs developing into planktotrophic trochophores, whereas the other morph deposits large eggs developing into lecithotrophic (or facultatively planktotrophic) larvae. *Boccardia proboscidea*[141] deposits egg masses with each egg capsule containing many eggs, some of which develop into larvae which hatch as planktotrophic larvae, whereas other embryos feed on nurse eggs and hatch as advanced larvae or small juveniles. The intracapsular trochophora larvae are apparently morphologically differentiated to the stage where feeding of the yolk granules should be possible, but feeding was not observed. A similar type of development is found in *Pygospio elegans*[142] (Figure 11). It must be assumed that the fertilized eggs all contain the genetic information needed for the organization of a feeding trochophore, with only a small genetic switch deciding whether this program or the abbreviated program for direct development becomes activated.

Similar developmental variation is found in the gastropod *Alderia,* where *A. modesta* shows only planktotrophic development, whereas *A. willowi* shows poecilogony; in some cases, the same individual was observed to deposit only egg masses with embryos hatching as lecithotrophic larvae just after collection, but after 20 days in captivity, 60% of the larvae from an egg mass were planktotrophic [143].

The occurrence of Hox genes may also indicate that the ancestral eumetazoan was a radial (or only slightly bilateral) gastrula. Cnidarians and (ctenophores) lack the long bilaterian Hox cluster. In the protostomes, Hox gene expression is documented in detail in the annelid *Nereis*, where Hox genes are not expressed in the prostomium and peristomium [144]. A similar expression pattern is seen in the gastropod *Haliotis*[145]. In the Arthropoda, Hox gene expression is similarly absent in the development of the supposedly homologous ocular and antennal/cheliceral segments [146]. This could indicate that a more radial-type larva was ancestral and that the bilateral elongation of the body with the collateral Hox cluster is a later addition related to the shift from an ancestrally radial pelagic ancestor to a bilateral organism with a benthic adult life style.

The diagram showing “progressive evolutionary interpolation of larval development …” [10] (Figure 4; reproduced in several later publications) is pure speculation without any basis in direct observations or considerations about adaptation. These scattered observations all indicate that the eumetazoan ancestor had indirect development with a planktotrophic larva.

#### Scenarios: Functional morphology and evolution

It goes without saying that evolution is the result of descent with modification and natural selection. This implies that all organisms/organs/structures have evolved continuously through small steps, with each new step giving some advantage. Non-adaptive modifications will be selected against, and proposed evolutionary series where only the end point is functional/advantageous must be rejected as orthogenesis [147]. Accordingly, all scenarios must be checked for two properties: 1) Have all the proposed ancestors and the stages between them and their descendants been able to feed, move and reproduce, and 2) Did each small step in the proposed evolution confer an advantage, so that it could be selected for. In this section, I will discuss the scenarios implied in the two main theories for the evolution of the eumetazoan life cycles with these basal principles in mind.

The basal part of the metazoan tree is still discussed, but the Eumetazoa clearly comprise Cnidaria and Bilateria and the Bilateria comprise Protostomia and Deuterostomia [22,148]. The positions of Ctenophora and Acoelomorpha are uncertain, but they will not be discussed here.

*The terminal addition theory* proposes that the eumetazoan ancestor was a holopelagic feeding gastraea and that a benthic stage was added to the life cycles in various lineages.

The evolution of the cnidarians from a holopelagic gastraea was a simple addition of a sessile adult stage, probably facilitated by the evolution of nematocysts. All anthozoans and most medusozoans have indirect development and planktotrophic gastrula larva are found in several anthozoans. The ancestral life cycle was most likely indirect with a feeding larva.

**Figure 12:**
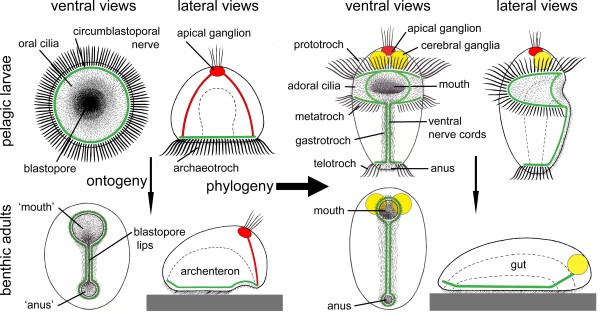
**The trochaea theory.** The upper part of the left side shows the ancestral trochaea. The left side shows the life cycle of a trochaea which has added a creeping, benthic stage to its life cycle and established a functional tube-shaped gut by lateral compression of the lateral blastopore lips. The right side shows the life cycle of a protostomian ancestor which has developed a permanent tube-shaped gut by fusion of the lateral blastopore lips and differentiated the archaeotroch into the anterior proto- and metatroch around the mouth and the telotroch around the anus; prototroch and metatroch plus the adoral ciliary zone forms a downstream-collecting ciliary system. From [75].

The trochaea-theory for the evolution of the protostomes (Figure 12), in particular of the spiralians, from the gastraea has been discussed in detail elsewhere [75]. It can be summarized in a number of steps: 1) The eumetazoan (neuralian) ancestor was a gastraea which developed a circumblastoporal ring of locomotory cilia which became specialized as compound cilia functioning as a downstream-collecting system (the archaeotroch) in contact with a circumblastoporal nerve; this was now a trochaea (Figure 12, upper left). The posterior position of the archaeotroch maximizes the efficiency of the ciliary beat [149] and corresponds to the position of the ring of compound cilia at the posterior pole of the (non-feeding) larvae some of demosponges [150]. A small apical organ was connected to the circumblastoporal nerve ring and probably regulated the beat of the archaeotroch. 2) An adult benthic stage was added and lost the compound cilia because the animal began creeping and feeding on deposited material. The ciliary field around the blastopore was used for transporting sediment particles to the gut (as in the gastraea/trochaea). The apical organ of the creeping adult moved towards a new anterior pole, and lateral blastopore closure created a through gut with anterior mouth and posterior anus (amphistomy) (Figure 12, lower left). 3) The lateral blastopore closure became permanent already in the larva (adultation [3]), and the archaeotroch became divided into an anterior, perioral part and a posterior, perianal ring (telotroch). The anterior ring expanded in a pair of elongate lateral loops with an anterior (prototroch) and posterior (metatroch) part. This was now a trochophora larva (Figure 12, upper right). 4) The apical organ of the trochaea was lost in the adult, whereas the periblastoporal nerve became differentiated into an oral loop (with an anterior part becoming incorporated in the brain), a pair of nerve cords along the fused blastopore lips, and a small perianal loop. A pair of cerebral ganglia differentiated from areas lateral to the apical organ and became the main part of the adult brain (Figure 12, lower right). These proposed evolutionary steps are all gradual and each step appears to provide some advantage.

No reasonable scenario has been proposed for the evolution of phoronids, brachiopods and bryozoans. Ecdysozoans lack ciliated outer epithelia and primary larvae and are obviously descended from a spiralian stem lineage with ciliated epithelia [22].

There seems to be no scenario for the origin of the deuterostomes, but it seems undisputed that the ancestral ambulacrarian had indirect development with a dipleurula larva [115]. The ancestral cephalochordate probably had a ciliated feeding larva, but the life cycle of the ancestral “olfactor” (the latest common ancestor of urochordates and vertebrates) remains obscure.

*The intercalation theories* propose that the planktotrophic larvae are stages intercalated into an ancestral direct development. Several variations of this idea have been proposed for more than a century. The planuloid-acoeloid theory [151], which derives the bilaterians from a non-feeding planuloid ancestor via a compact acoel-like form, was forcefully advocated by [152] and followed more or less tacitly in many textbooks. In the original form of the intercalation theory the proposed ancestor, the planula, had no gut and could therefore probably not feed; all non-feeding planula larvae develop from eggs with much yolk, and no adult free-living organisms of this organization is known. However, most recent proponents of this theory now agree that ancestor had a sack-shaped gut (and was therefore a gastraea) [5,10,153]. So the difference between the two theories is more about the life style of the ancestor. The terminal addition theory proposes a pelagic, planktotrophic gastrula and the intercalation theories a benthic, deposit-feeding, possibly bilateral ancestor (Figure 1).

There are only few attempts to visualize the intercalation theory for the bilaterians. Evolution of a pelagic larva from the benthic juvenile has been proposed as an adaptation to enhance dispersal, which appears reasonable. The evolution of a ring of compound cilia either at an anterior or a posterior ridge, as that also seen in some sponge larvae, enhances swimming [149], and this explanation is common to both the terminal addition and the intercalation theories. In the intercalation theories, the larva could be a lecithotrophic trochophore with only a prototroch or a metatroch.

**Figure 13:**
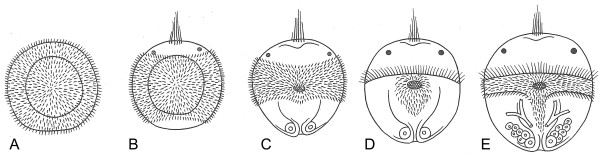
**Successive stages of the evolutionary transformation of the atrochal larva into a trochophore. A**, A completely ciliated larva. **B**, Larva with an apical tuft and an equatorial band of cilia. **C**, Larva with mouth and anus. **D**, Larva with the ciliation restricted to a prototroch and a perioral ciliation. **E**, Larva with prototroch, adoral ciliary zone and metatroch. Modified from [154].

For the spiralians, most of the intercalation theories propose that the planktotrophic trochophora larvae evolved many times by specializations of an ancestral uniformly ciliated non-feeding planula through a larva with a prototroch to the feeding trochophore. The diagram of [154] illustrates one version of this idea (Figure 13). The specializations of the ciliation in steps A-D, with the establishment of a prototroch, could be an adaptation to more powerful swimming, and the development of the mouth (and anus?) already in the larva could be a simple expression of adult characters already in the larva. However, evolution of a metatroch with the ciliary beat opposing that of the prototroch would hamper swimming and appears highly improbable. The whole evolution of the downstream-collecting system (Figure 13 D-E) appears as an example of orthogenesis because the structure will not be functional until fully formed. Hejnol A, 2007, Henry JQ, 2007 [11,12] suggested that the metatroch evolved through co-option of the posterior row of cells of the prototroch. However, the origin of a band of compound cilia with a beat opposite that of the prototroch through a split from the prototroch appears highly unlikely for two reasons: 1) During the evolution, the two bands would have been beating towards each other, and this can have no adaptive value before the whole downstream-collecting system has become functioning. 2) The origin of the metatroch through a split of the prototroch would imply a reversal of the beat in the metatroch, and ciliary reversals have never been observed in bands of compound cilia. The origin of the secondary trochoblasts and the metatroch from cells of the second micromere quartet around the blastopore is an integral part of the trochaea theory. I do not know any scenario for the origin of the pelago-benthic life cycle of the ambulacrarians.

It appears that only the terminal addition theory provides scenarios explaining the origin of the indirect life cycles with planktotrophic larvae which are in accordance with the principles of evolution through natural selection and adaptation.

## Conclusions

The fossil record contains no information about the type of life cycle of the Precambrian eubilaterians. Some Early Cambrian fossils demonstrate the presence of planktotrophic larvae in molluscs and brachiopods. A few studies of evolution in lineages of gastropods and echinoderms demonstrate multiple origin of non-planktotrophic development in clades with planktotrophic larvae, but there seems to be no example of an evolution in the opposite direction. Studies of the development of Living species shows good examples of multiple evolution of non-planktotrophic development in groups with predominantly planktotrophic development, but the picture is not unambiguous. Many papers simply conclude that the planktotrophic larvae occur so scattered in the metazoans that multiple, convergent evolution seems probable. The few cladistic analyses suffer from the misconception that a gain of a complicated ciliary feeding structure is of the same weight as a loss of the structure. There seems to bee no direct study of the genetic information involved, but the elegant studies of the evolution of tailless (anuran) larvae of ascidians demonstrate that the silencing of a single gene is responsible for the loss of the elongation of the tissues of the tail. However, the differentiation of the tissues characteristic of the chordates evolved in the chordate ancestors and has been retained in the anuran larvae.

The modern version of the intercalation theory proposes a gastrula stage in the ancestral eumetazoan, so both this and the terminal-addition theory can accommodate the cnidarians which added a sessile stage to the planktonic stage. The ancestral bilaterian was probably a gastraea too, the difference between the two theories being whether the gastraea was pelagic and planktotrophic (terminal addition theory) or benthic and deposit-feeding (intercalation theory).

**Figure 14:**
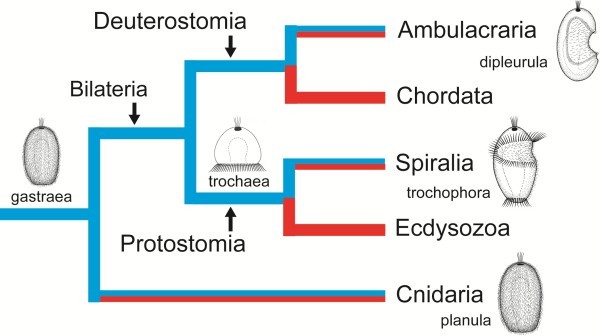
**Occurrence of life cycle types in the Eumetazoa (Neuralia).** Clades with planktotrophic (blue) and lecithotrophic/direct development (red) in major eumetazoan (neuralian) clades; blue/red clades indicate lineages of both types. The characteristic larval types, gastrula, trochophora and dipleurula are indicated. The ancestors of Eumetazoa (gastraea) and Protostomia (trochaea) are indicated, but the ancestors of Bilateria and Deuterostomia have not been envisaged.

The terminal addition theory scenarios explain the life cycles of most protostome phyla, but provide no scenario for the deuterostomes. The trochaea theory for the origin of the spiralians is in full accordance with the Darwinian principle of evolution through gradualism and adaptation. It is in good accordance with the many examples of evolution of non-planktotrophic development within smaller clades which ancestrally have life cycles with planktotrophic larvae. The planktotrophic larvae have been modified in many fascinating ways in the various lineages, and lecithotrophic larvae or direct development has evolved in many other lineages. The speciation event which lead towards lecithotrophy or direct development in one of the two sister species may have happened quite recently, so that species pairs with one species with indirect and one (or a few) with direct development are now seen. More ancient speciation events produced ancestors of larger clades with direct development, from genera or families all the way to the largest clades of the animal kingdom, such as Ecdysozoa and Chordata (Figure 14). It appears that planktotrophy can be regained after a period if the genes organizing the feeding structures have been retained. However, non-functioning genes are probably only retained for shorter periods, so re-evolution of feeding structures are only likely to happen after shorter evolutionary periods of non-feeding [94].

The intercalation theory provides no adaptation-based scenario for the origin of the planktotrophic larvae.

It can only be concluded that the ancestral eumetazoan/neuralian was a holopelagic, planktotrophic gastraea and that indirect (pelago-benthic) life cycles evolved in a few lineages through terminal addition of adult stages with the planktotrophic stages retained as larvae. In the cnidarians, the benthic stage was sessile, whereas the benthic stage in the bilaterians was vagile/creeping. It remains uncertain whether the vagile/creeing stage evolved in the last common ancestor of all bilaterians or separately in Protostomia and Deuterostomia. Non-feeding larvae and direct development developed in many clades (Figure 14). This implies that the larval types are important phylogenetic markers and not just isolated specializations with numerous complex homoplasies.

## Competing interests

The author declare that he has no competing interests.

## References

[B1] Wray GA McEdward L Evolution of larvae and developmental modes Ecology of Marine Invertebrate Larvae 1995 Boca Raton: CRC Press 413 447

[B2] Peterson KJ Davidson EH Regulatory evolution and the origin of the bilaterians Proc Natl Acad Sci U S A 2000 97 9 4430 4433 10.1073/pnas.97.9.4430 10781037 PMC34315

[B3] Jägersten G Evolution of the Metazoan Life Cycle 1972 London: Academic Press

[B4] Arenas-Mena C Indirect development, transdifferentiation and the macroregulatory evolution of metazoans Phil Trans R Soc B 2010 365 1540 653 669 10.1098/rstb.2009.0253 20083640 PMC2817142

[B5] Raff RA Origins of the other metazoan body plans: the evolution of larval forms Phil Trans R Soc B 2008 363 1496 1473 1479 10.1098/rstb.2007.2237 18192188 PMC2614227

[B6] Valentine JW Collins AG The significance of moulting in Ecdysozoan evolution Evol Dev 2000 2 3 152 156 10.1046/j.1525-142x.2000.00043.x 11252571

[B7] Gharbiah M Nakamoto A Nagy L Analysis of ciliary band formation in the mollusc *Ilyanassa obsoleta* Dev Genes Evol 2013 223 225 235 10.1007/s00427-013-0440-1 23592252

[B8] Haeckel E Die Gastraea-Theorie, die phylogenetische Classification des Thierreichs und die Homologie der Keimblätter Jena Z Naturw 1874 8 1 55

[B9] Hatschek B Lehrbuch der Zoologie, 3. Lieferung (pp 305–432) 1891 Gustav Fischer: Jena

[B10] Sly BJ Snoke MS Raff RA Who came first - larvae or adults? Origins of bilaterian metazoan larvae Int J Dev Biol 2003 47 623 632 14756338

[B11] Hejnol A Martindale MQ Henry JQ High-resolution fate map of the snail *Crepidula fornicata*: The origins of ciliary bands, nervous system, and muscular elements Dev Biol 2007 305 1 63 76 10.1016/j.ydbio.2007.01.044 17346693

[B12] Henry JQ Hejnol A Perry KJ Martindale MQ Homology of ciliary bands in spiralian trochophores Integr Comp Biol 2007 47 6 865 871 10.1093/icb/icm035 21669765

[B13] Rouse GW Trochophore concepts: ciliary bands and the evolution of larvae in spiralian Metazoa Biol J Linn Soc 1999 66 4 411 464 10.1111/j.1095-8312.1999.tb01920.x

[B14] Haeckel E Natürliche Schöpfungsgeschichte 1868 Berlin: Georg Reimer

[B15] Hadzi J An attempt to reconstruct the system of animal classification Syst Zool 1953 2 145 154 10.2307/2411558

[B16] Baldauf SL The deep roots of eukaryotes Science 2003 300 5626 1703 1706 10.1126/science.1085544 12805537

[B17] Rieger R The biphasic life cycle—A central theme of metazoan evolution Am Zool 1994 34 4 484 491

[B18] Dewel RA Colonial origin for Eumetazoa: major morphological transitions and the origin of bilaterian complexity J Morphol 2000 243 1 35 74 10.1002/(SICI)1097-4687(200001)243:1<35::AID-JMOR3>3.0.CO;2-# 10629096

[B19] Martynov A Ontogeny, systematics, and phylogenetics: Perspectives of future synthesis and a new model of the evolution of Bilateria Biol Bull (Woods Hole) 2012 39 5 393 401 23136734

[B20] Mikhailov KV Konstantinova AV Nikitin MA Troshin PV Rusin LY Lyubetsky VA Panchin YV Mylnikov AP Moroz LL Kumar S The origin of Metazoa: a transition from temporal to spatial cell differentiation Bioessays 2009 31 7 758 768 10.1002/bies.200800214 19472368

[B21] Nielsen C Six major steps in animal evolution: are we derived sponge larvae? Evol Dev 2008 10 2 241 257 10.1111/j.1525-142X.2008.00231.x 18315817

[B22] Nielsen C Animal Evolution: Interrelationships of the Living Phyla 2012 3 Oxford: Oxford University Press

[B23] Bütschli O Bemerkungen zur Gastraeatheorie Morph Jb 1884 9 415 427

[B24] Degnan SM Degnan BM The origin of the pelagobenthic metazoan life cycle: what's sex got to do with it? Integr Comp Biol 2006 46 6 683 690 10.1093/icb/icl028 21672778

[B25] Sperling EA Vinther J A placozoan affinity for *Dickinsonia* and the evolution of late Proterozoic metazoan feeding modes Evol Dev 2010 12 2 201 209 10.1111/j.1525-142X.2010.00404.x 20433459

[B26] Raikova EV Life cycle, cytology, and morphology of *Polypodium hydriforme*, a coelenterate parasite of the eggs of the acipenseriform fishes J Parasitol 1994 80 1 22 7905920

[B27] Balfour FM Larval forms: their nature, origin, and affinities Q J Microsc Sci, N S 1880 20 80 381 407

[B28] Willman S Morphology and wall ultrastructure of leiosphaeric and acanthomorphic acritarchs from the Ediacaran of Australia Geobiology 2009 7 1 8 20 10.1111/j.1472-4669.2008.00178.x 19200142

[B29] Budd GE The earliest fossil record of the animals and its significance Phil Trans R Soc B 2008 363 1496 1425 1434 10.1098/rstb.2007.2232 18192192 PMC2614223

[B30] Xiao S Zhang Y Knoll AH Three-dimensional preservation of algae and animal embryos in a Neoproterozoic phosphorite Nature 1998 391 6667 553 558 10.1038/35318

[B31] Hagadorn JW Xiao S Donoghue PCJ Bengtson S Gostling NJ Pawlowska M Raff EC Raff RA Turner FR Chongyu Y Cellular and subcellular structure of Neoproterozoic animal embryos Science 2006 314 5797 291 294 10.1126/science.1133129 17038620

[B32] Bailey JV Joye SB Kalanetra KM Flood BE Corsetti FA Evidence of giant sulphur bacteria in Neoproterozoic phosphorites Nature 2007 445 7124 198 201 10.1038/nature05457 17183268

[B33] Huldtgren T Cunningham JA Yin C Stampanoni M Marone F Donoghue PCJ Bengtson S Fossilized nuclei and germination structures identify Ediacaran “animal embryos” as encysting protists Science 2011 334 6063 1696 1699 10.1126/science.1209537 22194575

[B34] Chen J-Y Bottjer DJ Oliveri P Dornbos SQ Gao F Ruffins S Chi H Li C-W Davidson EH Small bilaterian fossils from 40 to 55 million years before the Cambrian Science 2004 305 5681 218 222 10.1126/science.1099213 15178752

[B35] Chen J-Y Bottjer DJ Li G Hadfield MG Gao F Cameron AR Zhang C-Y Xian D-C Tafforeau P Liao X Complex embryos displaying bilaterian characters from Precambrian Doushantuo phosphate deposits, Weng'an, Guizhou, China Proc Natl Acad Sci U S A 2009 106 45 19056 19060 10.1073/pnas.0904805106 19858483 PMC2776410

[B36] Petryshyn VA Bottjer DJ Chen J-Y Gao F Petrographic analysis of new specimens of the putative microfossil *Vernanimalcula guizhouena* (Doushantuo Formation, South China) Precambrian Res 2013 225 58 66

[B37] Bengtson S Cunningham JA Yin C Donoghue PCJ A merciful death for the “earliest bilaterian, Vernanimalcula Evol Dev 2012 14 5 421 427 10.1111/j.1525-142X.2012.00562.x 22947315

[B38] Chen J-Y Oliveri P Li C-W Zhou G-Q Gao F Hagadorn JW Peterson KJ Davidson EH Precambrian animal diversity: putative phosphatized embryos from the Doushantuo Formation of China Proc Natl Acad Sci U S A 2000 97 9 4457 4462 10.1073/pnas.97.9.4457 10781044 PMC18256

[B39] Zhao YUE Bengtson S Embryonic and post-embryonic development of the Early Cambrian cnidarian *Olivooides* Lethaia 1999 32 2 181 195

[B40] Chen F Dong X The internal structure of Early Cambrian fossil embryo *Olivooides* revealed in the light of synchrotron X-ray tomographic microscopy Chin Sci Bull 2008 53 24 3860 3865 10.1007/s11434-008-0452-9

[B41] Dong X-P Cunningham JA Bengtson S Thomas C-W Liu J Stampanoni M Donoghue PCJ Embryos, polyps and medusae of the Early Cambrian scyphozoan *Olivooides* Proc R Soc Lond B 2013 280 1757 doi:http://dx.doi.org/10.1098/rspb.2013.0071, http://rspb.royalsocietypublishing.org/content/280/1757/20130071.abstract 10.1098/rspb.2013.0071 PMC3619488 23446532

[B42] Freeman G The rise of bilaterians Hist Biol 2009 21 1–2 99 114

[B43] Fedonkin MA Simonetta A Ivantsov AY Vickers-Rich P, Komarower P New data on *Kimberella*, the Vendian mollusc-like organism (White Sea region, Russia): palaeoecological and evolutionary implications The Rise and Fall of the Ediacaran Biota 2007 London: The Geological Society 157 179

[B44] Hou X-G Aldridge RJ Bergström J Siveter DJ Siveter DJ Feng X-H The Cambrian Fossils of Chengjiang, China 2004 Malden, MA: Blackwell

[B45] Briggs DEG Erwin DH Collier FJ The Fossils of the Burgess Shale 1994 Washington: Smithsonian Institution Press

[B46] Marshall DJ Krug PJ Kupriyanova EK Byrne M Emlet RB The biogeography of marine invertebrate life histories Annu Rev Ecol Evol Syst 2012 43 1 97 114 10.1146/annurev-ecolsys-102710-145004

[B47] Maloof AC Porter SM Moore JL Dudás FÖ Bowring SA Higgins JA Fike DA Eddy MP The earliest Cambrian record of animals and ocean geochemical change Geol Soc Am Bull 2010 122 11–12 1731 1774

[B48] Mus MM Palacios T Jensen S Size of the earliest mollusks: Did small helcionellids grow to become large adults? Geology 2008 36 2 175 178 10.1130/G24218A.1

[B49] Vinther J Sperling EA Briggs DEG Peterson KJ A molecular palaeobiological hypothesis for the origin of aplacophoran molluscs and their derivation from chiton-like ancestors Proc R Soc Lond B 2012 279 1732 1259 1268 10.1098/rspb.2011.1773 PMC3282371 21976685

[B50] Vendrasco MJ Checa AG Kouchinsky AV Shell microstructure of the early bivalve *Pojetaia* and the independent origin of nacre within the Mollusca Palaeontology 2011 54 4 825 850 10.1111/j.1475-4983.2011.01056.x

[B51] Ockelmann KW Cox LR, Peake JF Developmental types in marine bivalves and their distribution along the Atlantic coast of Europe Proceedings of the 1st European Malacological Congress 1965 London: Choncological Society of Great Britan and Ireland and Malacological Society of London 25 35

[B52] Nützel A Lehnert O Frýda J Origin of planktotrophy—evidence from early molluscs: a response to Freeman and Lundelius Evol Dev 2007 9 4 313 318 10.1111/j.1525-142X.2007.00166.x 17651353

[B53] Runnegar B No evidence for planktotrophy in Cambrian molluscs Evol Dev 2007 9 4 311 312 10.1111/j.1525-142X.2007.00165.x 17651352

[B54] Freeman G Lundelius JW Origin of planktotrophy—evidence from early molluscs: a response to Nützel et al. (2006) Evol Dev 2007 9 4 307 310 10.1111/j.1525-142X.2007.00164.x 17651351

[B55] Frýda J Talent JA Phylogeny of Palaeozoic gastropods Inferred from their ontogeny Earth and Life International Year of Planet Earth, Part II 2012 Netherlands: Springer 395 435

[B56] Runnegar B Bentley C Anatomy, ecology and affinities of the Australian Early Cambrian bivalve *Pojetaia runnegari* Jell J Paleont 1983 57 1 73 92

[B57] Hansen TA Modes of larval development in Early Tertiary neogastropods Paleobiology 1982 8 4 367 377 10.1126/science.220.4596.501 17816221

[B58] Holmer LE Skovsted CB Larsson C Brock GA Zhang Z First record of a bivalved larval shell in Early Cambrian tommotiids and its phylogenetic significance Palaeontology 2011 54 2 235 239 10.1111/j.1475-4983.2010.01030.x

[B59] Smith AB Deuterostomes in a twist: the origins of a radical new body plan Evol Dev 2008 10 4 493 503 10.1111/j.1525-142X.2008.00260.x 18638326

[B60] Cunningham JA Jeffery Abt CH Coordinated shifts to non-planktotrophic development in spatangoid echinoids during the Late Cretaceous Biol Lett 2009 5 5 647 650 10.1098/rsbl.2009.0302 19515650 PMC2781954

[B61] Emlet RB Apical skeletons of sea urchins (Echinodermata: Echinoidea): Two methods for inferring mode of larval development Paleobiology 1989 15 3 223 254

[B62] Jeffery CH Dawn of echinoid nonplanktotrophy: Coordinated shifts in development indicate environmental instability prior to the K-T boundary Geology 1997 25 11 991 994 10.1130/0091-7613(1997)025<0991:DOENCS>2.3.CO;2

[B63] Rouse GW The epitome of hand waving? Larval feeding and hypotheses of metazoan phylogeny Evol Dev 2000 2 4 222 233 10.1046/j.1525-142x.2000.00063.x 11252565

[B64] Rouse GW Bias? What bias? The evolution of downstream larval-feeding in animals Zool Scr 2000 29 3 213 236 10.1046/j.1463-6409.2000.00040.x

[B65] Rouse GW Polychaetes have evolved feeding larvae numerous times Bull Mar Sci 2000 67 1 391 409

[B66] Riisgård HU Nielsen C Larsen PS Downstream collecting in ciliary suspension feeders: the catch-up principle Mar Ecol Prog Ser 2000 207 33 51

[B67] Dalyell JG Rare and Remarkable Animals of Scotland 1847 London: John van Voorst Volume 1.

[B68] Schwarz J Weis V Potts D Feeding behavior and acquisition of zooxanthellae by planula larvae of the sea anemone *Anthopleura elegantissima* Mar Biol 2002 140 3 471 478 10.1007/s00227-001-0736-y

[B69] Tranter PRG Nicholson DN Kinchington D A description of spawning and post-gastrula development of the cool temperate coral, *Caryophyllia smithi* J Mar Biol Assoc UK 1982 62 04 845 854 10.1017/S0025315400070387

[B70] Schwarz JA Krupp DA Weis VM Late larval development and onset of symbiosis in the scleractinian coral *Fungia scutaria* Biol Bull (Woods Hole) 1999 196 1 70 79 10.2307/1543169 25575388

[B71] Kayal E Roure B Philippe H Collins AG Lavrov DV Cnidarian phylogenetic relationships as revealed by mitogenomics BMC Evol Biol 2013 13 1 5 10.1186/1471-2148-13-5 23302374 PMC3598815

[B72] Hatschek B Studien über Entwicklungsgeschichte der Anneliden Arb Zool Inst Univ Wien 1878 1 277 404

[B73] Woltereck R Wurm"kopf", Wurmrumpf und Trochophora Zool Anz 1904 28 273 322

[B74] Nielsen C Larval ciliary bands and metazoan phylogeny Fortschr Zool Syst Evolutionsforsch 1979 1 178 184

[B75] Nielsen C How to make a protostome Invertebr Syst 2012 26 1 25 40 10.1071/IS11041

[B76] Struck TH Paul C Hill N Hartmann S Hosel C Kube M Lieb B Meyer A Tiedemann R Purschke G Phylogenomic analyses unravel annelid evolution Nature 2011 471 7336 95 98 10.1038/nature09864 21368831

[B77] Emlet RB Strathmann RR Wilson WH Functional consequences of simple cilia in the mitraria of Oweniids (an anomalous larva of an anomalous polychaete) and comparisons with other larvae Reproduction and Development of Marine Invertebrates 1994 Baltimore: Johns Hopkins University Press 143 157

[B78] Smart TI Von Dassow G Unusual development of the mitraria larva in the polychaete *Owenia collaris* Biol Bull (Woods Hole) 2009 217 3 253 268 10.1086/BBLv217n3p253 20040750

[B79] Strathmann RR Jahn TL Fonseca JRC Suspension feeding by marine invertebrate larvae: clearance of particles by ciliated bands of a rotifer, pluteus, and trochophore Biol Bull (Woods Hole) 1972 142 3 505 519 10.2307/1540326

[B80] Pernet B Strathmann RR Opposed ciliary bands in the feeding larvae of sabellariid annelids Biol Bull (Woods Hole) 2011 220 3 186 198 10.1086/BBLv220n3p186 21712227

[B81] Miner BG Sanford E Strathmann RR Pernet B Emlet RB Functional and evolutionary implications of opposed bands, big mouths, and extensive oral ciliation in larval opheliids and echiurids (Annelida) Biol Bull (Woods Hole) 1999 197 1 14 25 10.2307/1542993 28296496

[B82] Kudenov JD The reproductive biology of Eurythoe complanata (Pallas, 1766), (Polychaeta: Amphinomidae) Ph.D. Thesis 1974 Tucson: University of Arizona

[B83] Pernet B McArthur L Feeding by larvae of two different developmental modes in *Streblospio benedicti* (Polychaeta: Spionidae) Mar Biol 2006 149 803 811 10.1007/s00227-006-0266-8

[B84] Hansen B Aspects of feeding, growth and stage development by trochophora larvae of the boreal polychaete *Mediomastus fragile* (Rasmussen) (Capitellidae) J Exp Mar Biol Ecol 1993 166 273 288 10.1016/0022-0981(93)90224-C

[B85] Hatschek B Ueber Entwicklungsgeschichte von *Echiurus* und die systematische Stellung der Echiuridae (Gephyrei chaetiferi) Arb Zool Inst Univ Wien 1880 3 45 78

[B86] McDougall C Chen W-C Shimeld S Ferrier D The development of the larval nervous system, musculature and ciliary bands of *Pomatoceros lamarckii* (Annelida): heterochrony in polychaetes Front Zool 2006 3 1 16 10.1186/1742-9994-3-16 17032451 PMC1615870

[B87] Pernet B Persistent ancestral feeding structures in nonfeeding annelid larvae Biol Bull Woods Hole 2003 205 295 307 10.2307/1543293 14672984

[B88] Sutton MD Briggs DEG Siveter DJ Siveter DJ Sigwart JD A Silurian armoured aplacophoran and implications for molluscan phylogeny Nature 2012 490 7418 94 97 10.1038/nature11328 23038472

[B89] Page LR Ferguson SJ The other gastropod larvae: Larval morphogenesis in a marine neritimorph J Morphol 2013 274 412 428 10.1002/jmor.20103 23192866

[B90] Duda TF Palumbi SR Developmental shifts and species selection in gastropods Proc Natl Acad Sci U S A 1999 96 18 10272 10277 10.1073/pnas.96.18.10272 10468598 PMC17878

[B91] Reid DG Systematics and evolution of Littorina 1996 London: Ray Society

[B92] Reid DG The comparative morphology, phylogeny and evolution of the gastropod family Littorinidae Phil Trans R Soc B 1989 324 1220 1 110 10.1098/rstb.1989.0040

[B93] Lieberman BS Allmon WD Eldredge N Levels of selection and macroevolutionary patterns in the turritellid gastropods Paleobiology 1993 19 2 205 215

[B94] Collin R Chaparro OR Winkler F Véliz D Molecular phylogenetic and embryological evidence that feeding larvae have been reacquired in a marine gastropod Biol Bull (Woods Hole) 2007 212 2 83 92 10.2307/25066586 17438201

[B95] Collin R Phylogenetic effects, the loss of complex characters, and the evolution of development in calyptraeid gastropods Evolution 2004 58 7 1488 1502 15341151 10.1111/j.0014-3820.2004.tb01729.x

[B96] Fioroni P Zur Morphologie und Embryogenese des Darmtraktes und der transistorischen Organe bei Prosobranchiern (Mollusca, Gastropoda) Rev Suisse Zool 1966 73 621 876 5974455

[B97] Hadfield MG Iaea DK Velum of encapsulated veligers of *Petaloconchus* (Gastropoda), and the problem of Re-evolution of planktotrophic larvae Bull Mar Sci 1989 45 2 377 386

[B98] McEdward LR Janies DA Life cycle evolution in asteroids: What is a larva? Biol Bull (Woods Hole) 1993 184 3 255 268 10.2307/1542444 29300539

[B99] Sharma PP González VL Kawauchi GY Andrade SCS Guzmán A Collins TM Glover EA Harper EM Healy JM Mikkelsen PM Phylogenetic analysis of four nuclear protein-encoding genes largely corroborates the traditional classification of Bivalvia (Mollusca) Mol Phylogenet Evol 2012 65 1 64 74 10.1016/j.ympev.2012.05.025 22659514

[B100] Cragg SM Taylor J The phylogenetic significance of some anatomical features of bivalve larvae Origin and Evolutionary Radiation of the Mollusca 1996 Oxford: Oxford Univ. Press 371 380

[B101] Nielsen C Trochophora larvae: cell-lineages, ciliary bands, and body regions. 1. Annelida and Mollusca J Exp Zool (Mol Dev Evol) 2004 302B 1 35 68 10.1002/jez.b.20001 14760653

[B102] Jägersten G On the morphology and reproduction of entoproct larvae Zool Bidr Upps 1964 36 295 314

[B103] Nielsen C Entoproct life-cycles and the entoproct/ectoproct relationship Ophelia 1971 9 2 209 341 10.1080/00785326.1971.10430095

[B104] Nielsen C Worsaae K Structure and occurrence of cyphonautes larvae (Bryozoa, Ectoprocta) J Morphol 2010 271 9 1094 1109 10.1002/jmor.10856 20730922

[B105] Waeschenbach A Taylor PD Littlewood DTJ A molecular phylogeny of bryozoans Mol Phylogenet Evol 2012 62 2 718 735 10.1016/j.ympev.2011.11.011 22126903

[B106] Taylor PD Carboniferous and Permian species of the cyclostome bryozoan *Corynotrypa* Bassler, 1911 and their clonal propagation Bull Br Mus (Nat Hist), Geol 1985 38 359 372

[B107] Todd JA Herrera Cubilla A, Jackson JBC The central role of ctenostomes in bryozoan phylogeny Proceedings of the 11th International Bryozoology Association Conference 2000 Balboa, Panama: Smithsonian Tropical Research Institute 104 135

[B108] Nielsen C Ciliary filter-feeding structures in adult and larval gymnolaemate bryozoans Invertebr Biol 2002 121 3 255 261

[B109] Nielsen C Riisgård HU Tentacle structure and filter-feeding in *Crisia eburnea* and other cyclostomatous bryozoans, with a review of upstream-collecting mechanisms Mar Ecol Prog Ser 1998 168 163 186

[B110] Riisgård HU Manríquez P Filter-feeding in fifteen marine ectoprocts (Bryozoa): particle capture and water pumping Mar Ecol Prog Ser 1997 154 223 239

[B111] Strathmann RR Versatile ciliary behaviour in capture of particles by the bryozoan cyphonautes larva Acta Zool (Stockh) 2006 87 1 83 89 10.1111/j.1463-6395.2006.00224.x

[B112] Riisgård HU Methods of ciliary filter feeding in adult *Phoronis muelleri* (phylum Phoronida) and its free-swimming actinotroch larva Mar Biol 2002 141 75 87 10.1007/s00227-002-0802-0

[B113] Temereva E Malakhov V Filter feeding mechanism in the phoronid *Phoronopsis harmeri* (Phoronida, Lophophorata) Russ J Mar Biol 2010 36 2 109 116 10.1134/S1063074010020057

[B114] Zimmer RL Gilbert SF, Raunio AM Phoronids, brachiopods, and bryozoans, the lophophorates Embryology Constructing the Organism 1997 Sunderland: Sinauer Associates 279 305

[B115] Peterson KJ Cameron RA Davidson EH Bilaterian origins: Significance of new experimental observations Dev Biol 2000 219 1 1 17 10.1006/dbio.1999.9475 10677251

[B116] Byrne M Nakajima Y Chee FC Burke RD Apical organs in echinoderm larvae: insights into larval evolution in the Ambulacraria Evol Dev 2007 9 432 445 10.1111/j.1525-142X.2007.00189.x 17845515

[B117] Strathmann RR Time and extent of ciliary response to particles in a non-filtering feeding mechanism Biol Bull (Woods Hole) 2007 212 2 93 103 10.2307/25066587 17438202

[B118] Pisani D Feuda R Peterson KJ Smith AB Resolving phylogenetic signal from noise when divergence is rapid: A new look at the old problem of echinoderm class relationships Mol Phylogenet Evol 2012 62 1 27 34 10.1016/j.ympev.2011.08.028 21945533

[B119] Nakano H Hibino T Oji T Hara Y Amemiya S Larval stages of a living sea lily (stalked crinoid echinoderm) Nature 2003 421 158 160 12520300 10.1038/nature01236

[B120] Lacalli TC Ciliary bands in echinoderm larvae: evidence for structural homologies and a common plan Acta Zool (Stockh) 1993 74 127 133 10.1111/j.1463-6395.1993.tb01229.x

[B121] Kroh A Smith AB The phylogeny and classification of post-Palaeozoic echinoids J Syst Palaeontol 2010 8 2 147 212 10.1080/14772011003603556

[B122] Wray GA Parallel evolution of nonfeeding larvae in echinoids Syst Biol 1996 45 3 308 322 10.1093/sysbio/45.3.308

[B123] O'Loughlin PM Waters JM A molecular and morphological revision of genera of Asterinidae (Echinodermata: Asteroidea) Mem Mus Vic 2004 61 1 40

[B124] Raff RA Byrne M The active evolutionary lives of echinoderm larvae Heredity 2006 97 3 244 252 16850040 10.1038/sj.hdy.6800866

[B125] Nielsen C Hay-Schmidt A Development of the enteropneust *Ptychodera flava*: ciliary bands and nervous system J Morphol 2007 268 7 551 570 10.1002/jmor.10533 17469131

[B126] Nielsen C Some aspects of spiralian development Acta Zool (Stockh) 2010 91 1 20 28 10.1111/j.1463-6395.2009.00421.x

[B127] Nielsen C Minelli A, Fusco G Ontogeny of the spiralian brain Evolving Pathways: Key Themes in Evolutionary Developmental Biology 2008 Cambridge: Cambridge University Press 399 416

[B128] Richter S Loesel R Purschke G Schmidt-Rhaesa A Scholtz G Stach T Vogt L Wanninger A Brenneis G Doring C Invertebrate neurophylogeny: suggested terms and definitions for a neuroanatomical glossary Front Zool 2010 7 1 29 10.1186/1742-9994-7-29 21062451 PMC2996375

[B129] Martindale M Lee P The development of form: Causes and consequences of developmental reprogramming associated with rapid body plan evolution in the bilaterian radiation Biol Theory 2013 doi:http://dx.doi.org/10.1007/s13752-013-0117-z

[B130] Martin VJ Reorganization of the nervous system during metamorphosis of a hydrozoan planula Invertebr Biol 2000 119 3 243 253

[B131] Nielsen C Trochophora larvae: cell-lineages, ciliary bands and body regions. 2. Other groups and general discussion J Exp Zool (Mol Dev Evol) 2005 304B 5 401 447 10.1002/jez.b.21050 15915468

[B132] Gifondorwa DJ Leise EM Programmed cell death in the apical ganglion during larval metamorphosis of the marine mollusc *Ilyanassa obsoleta* Biol Bull Woods Hole 2006 210 109 120 10.2307/4134600 16641516

[B133] Nakano H Nakajima Y Amemiya S Nervous system development of two crinoid species, the sea lily *Metacrinus rotundus* and the feather star *Oxycomanthus japonicus* Dev Genes Evol 2009 219 11–12 565 576 20099068 10.1007/s00427-010-0317-5

[B134] Nielsen C Martinez P Patterns of gene expression: homology or homocracy? Dev Genes Evol 2003 213 3 149 154 12690454 10.1007/s00427-003-0301-4

[B135] Raff EC Popodi EM Kauffman JS Sly BJ Turner FR Morris VB Raff RA Regulatory punctuated equilibrium and convergence in the evolution of developmental pathways in direct-developing sea urchins Evol Dev 2003 5 5 478 493 10.1046/j.1525-142X.2003.03054.x 12950627

[B136] Raff EC Popodi EM Sly BJ Turner FR Villinski JT Raff RA A novel ontogenetic pathway in hybrid embryos between species with different modes of development Development 1999 126 9 1937 1945 10101127 10.1242/dev.126.9.1937

[B137] Love AC Lee AE Andrews ME Raff RA Co-option and dissociation in larval origins and evolution: the sea urchin larval gut Evol Dev 2008 10 1 74 88 10.1111/j.1525-142X.2007.00215.x 18184359

[B138] Hadfield KA Swalla BJ Jeffery WR Multiple origins of anural development in ascidians inferred from rDNA sequences J Mol Evol 1995 40 413 427 10.1007/BF00164028 7646666

[B139] Swalla BJ Jeffery WR Requirement of the *Manx* gene for expression of chordate features in a tailless ascidian larva Science 1996 274 5290 1205 1208 10.1126/science.274.5290.1205 8895472

[B140] Gibson G MacDonald K Dufton M Morphogenesis and phenotypic divergence in two developmental morphs of *Streblospio benedicti* (Annelida, Spionidae) Invertebr Biol 2010 129 4 328 343 10.1111/j.1744-7410.2010.00213.x

[B141] Gibson G Carver D Effects of extra-embryonic provisioning on larval morphology and histogenesis in *Boccardia proboscidea* (Annelida, Spionidae) J Morphol 2013 274 1 11 23 10.1002/jmor.20071 22965540

[B142] Rasmussen E Systematics and ecology of the Isefjord marine fauna Ophelia 1973 11 1 495 10.1080/00785326.1973.10430115

[B143] Krug PJ Poecilogony and larval ecology in the gastropod genus *Alderia* Am Malacol Bull 2007 23 1 99 111 10.4003/0740-2783-23.1.99

[B144] Kulakova M Bakalenko N Novikova E Cook C Eliseeva E Steinmetz PH Kostyuchenko R Dondua A Arendt D Akam M Hox gene expression in larval development of the polychaetes *Nereis virens* and *Platynereis dumerilii* (Annelida, Lophotrochozoa) Dev Genes Evol 2007 217 1 39 54 10.1007/s00427-006-0119-y 17180685

[B145] Hinman VF O'Brien EK Richards GS Degnan BM Expression of anterior *Hox* genes during larval development of the gastropod *Haliotis asinina* Evol Dev 2003 5 508 521 10.1046/j.1525-142X.2003.03056.x 12950629

[B146] Hughes CL Kaufman TC Hox genes and the evolution of the arthropod body plan Evol Dev 2002 4 6 459 499 10.1046/j.1525-142X.2002.02034.x 12492146

[B147] Mayr E The Growth of Biological Thought. Diversity, Evolution, and Inheritance 1982 Cambridge, MA: Harvard University Press

[B148] Edgecombe GD Giribet G Dunn CW Hejnol A Kristensen RM Neves RC Rouse G Worsaae K Sørensen MV Higher-level metazoan relationships: recent progress and remaining questions Org Divers Evol 2011 11 2 151 172 10.1007/s13127-011-0044-4

[B149] Emlet RB Functional constraints on the evolution of larval forms of marine invertebrates: experimental and comparative evidence Am Zool 1991 31 4 707 725

[B150] Woollacott RM Structure and swimming behavior of the larva of *Haliclona tubifera* (Porifera: Demospongiae) J Morphol 1993 218 3 301 321 10.1002/jmor.1052180306 29865486

[B151] Steinböck O Dougherty EC Origin and affinities of the lower Metazoa: the "acoeloid" ancestry of the Eumetazoa The Lower Metazoa 1963 Berkeley: Univ. California Press 40 54

[B152] Hyman LH The Invertebrates, vol. 2. Platyhelminthes and Rhynchocoela. The Acoelomate Bilateria 1951 New York: McGraw-Hill

[B153] Hejnol A Martindale MQ Acoel development supports a simple planula-like urbilaterian Phil Trans R Soc Lond B 2008 363 1493 1501 10.1098/rstb.2007.2239 18192185 PMC2614228

[B154] Ivanova-Kazas OM The origin and phylogenetic significance of the trochophoran larvae. 2. Evolutionary significance of the larvae of coelomate worms and molluscs (In Russian, English summary) Zool Zh 1985 64 650 660

